# Penetrance of Hypertrophic Cardiomyopathy in Sarcomere Protein Mutation Carriers

**DOI:** 10.1016/j.jacc.2020.06.011

**Published:** 2020-08-04

**Authors:** Massimiliano Lorenzini, Gabrielle Norrish, Ella Field, Juan Pablo Ochoa, Marcos Cicerchia, Mohammed M. Akhtar, Petros Syrris, Luis R. Lopes, Juan Pablo Kaski, Perry M. Elliott

**Affiliations:** a Barts Heart Centre, St. Bartholomew's Hospital, London, United Kingdom; b University College London Institute of Cardiovascular Science, London, United Kingdom; c Centre for Inherited Cardiovascular Diseases, Great Ormond Street Hospital, London, United Kingdom; d Health in Code S.L., Scientific Department, A Coruña, Spain; e Universidade da Coruña, GRINCAR (Cardiovascular Research Group), A Coruña, Spain

**Keywords:** cardiac magnetic resonance, ECG, echocardiogram, sex, sudden cardiac death, CMR, cardiac magnetic resonance, ECG, electrocardiogram, HCM, hypertrophic cardiomyopathy, ICD, implantable cardioverter-defibrillator, LV, left ventricular, P/LP, pathogenic/likely pathogenic, SCD, sudden cardiac death, SP, sarcomere protein

## Abstract

**Background:**

Predictive genetic screening of relatives of patients with hypertrophic cardiomyopathy (HCM) caused by sarcomere protein (SP) gene mutations is current standard of care, but there are few data on long-term outcomes in mutation carriers without HCM.

**Objectives:**

The aim of this study was to determine the incidence of new HCM diagnosis in SP mutation carriers.

**Methods:**

This was a retrospective analysis of adult and pediatric SP mutation carriers identified during family screening who did not fulfill diagnostic criteria for HCM at first evaluation.

**Results:**

The authors evaluated 285 individuals from 156 families (median age 14.2 years [interquartile range: 6.8 to 31.6 years], 141 [49.5%] male individuals); 145 (50.9%) underwent cardiac magnetic resonance (CMR). Frequency of causal genes was as follows: *MYBPC3* n = 123 (43.2%), *MYH7* n = 69 (24.2%), *TNNI3* n = 39 (13.7%), *TNNT2* n = 34 (11.9%), *TPM1* n = 9 (3.2%), *MYL2* n = 6 (2.1%), *ACTC1* n = 1 (0.4%), multiple mutations n = 4 (1.4%). Median follow-up was 8.0 years (interquartile range: 4.0 to 13.3 years) and 86 (30.2%) patients developed HCM; 16 of 50 (32.0%) fulfilled diagnostic criteria on CMR but not echocardiography. Estimated HCM penetrance at 15 years of follow-up was 46% (95% confidence interval [CI]: 38% to 54%). In a multivariable model adjusted for age and stratified for CMR, independent predictors of HCM development were male sex (hazard ratio [HR]: 2.91; 95% CI: 1.82 to 4.65) and abnormal electrocardiogram (ECG) (HR: 4.02; 95% CI: 2.51 to 6.44); *TNNI3* variants had the lowest risk (HR: 0.19; 95% CI: 0.07 to 0.55, compared to *MYBPC3*).

**Conclusions:**

Following a first negative screening, approximately 50% of SP mutation carriers develop HCM over 15 years of follow-up. Male sex and an abnormal ECG are associated with a higher risk of developing HCM. Regular CMR should be considered in long-term screening.

Provision of genetic testing to the relatives of patients with hypertrophic cardiomyopathy (HCM) caused by pathogenic/likely pathogenic (P/LP) variants in sarcomere protein (SP) genes is standard of care in clinical practice guidelines (1). Adoption of this recommendation has led to the identification of a growing number of individuals that carry SP gene P/LP variants in the absence of an overt cardiomyopathy on cardiac imaging who are offered lifelong follow-up. The evidence to support this strategy is, however, relatively weak (2, 3, 4, 5, 6, 7, 8), as most studies are limited by small cohort size, restriction to children (2,3), and the influence of founder SP gene variants (7,8). Ongoing screening of healthy SP P/LP variant carriers also poses a potentially unsustainable burden on health care providers and relatives alike. The primary aim of this study was to determine the incidence of a new HCM diagnosis in relatives with SP P/LP variants and to evaluate predictors of phenotype development. The secondary aim was to assess the incidence of major cardiovascular events in relatives carrying SP P/LP variants.

## Methods

This was a retrospective analysis of consecutive adult and pediatric SP P/LP variant carriers identified during family screening and who did not fulfill diagnostic criteria for HCM (1) at first clinical evaluation. All were evaluated between 1988 and October 2018 at the Inherited Cardiovascular Diseases units at The Heart Hospital, St Bartholomew’s Hospital, and Great Ormond Street Hospital in London. All underwent standard electrocardiography (ECG) and 2-dimensional echocardiography at 1 to 3 yearly intervals and cardiac magnetic resonance (CMR) imaging at physician’s discretion as previously described (9). The study conforms to the declaration of Helsinki (10) and the retrospective data collection was approved by the local ethics committee (reference 19/WS/0100).

### Genetics

All SP variants were reviewed and classified in January 2020 according to the American College of Medical Genetics and Genomics criteria (11). Only individuals with pathogenic or likely pathogenic variants in *MYBPC3, MYH7, TNNI3, TNNT2, MYL2, MYL3, TPM1,* or *ACTC1* were included in the study. Family members underwent genetic testing for SP variants identified in index cases using automated DNA sequencing methods on an ABI3130 genetic analyzer and BigDye Terminator v3.1 cycle sequencing chemistry and standard protocols (Applied Biosystems, Foster City, California).

### Study endpoints and definitions

The primary endpoint was a new diagnosis of HCM defined as left ventricular (LV) hypertrophy not explained solely by loading conditions (1). Specifically:

• Adults: LV wall thickness ≥13 mm on echocardiogram or CMR.
• Children: LV wall thickness more than 2 standard deviations (SDs) greater than the predicted mean (z-score >2).

The time of onset of the primary endpoint was defined as the earliest time a subject fulfilled the diagnostic criteria for HCM. Data were included up to the last available follow-up at the time the database was closed (June 30, 2019); subjects who did not reach the primary endpoint were censored at the time of their last evaluation. In subjects who died with no clinical diagnosis of HCM, the primary endpoint was adjudicated based on the postmortem results or censored at the time of their last evaluation in absence of a postmortem.

The incidence of major cardiovascular events (all-cause mortality, cardiac transplantation, aborted sudden cardiac death [SCD], and appropriate implantable cardioverter-defibrillator [ICD] shock) was determined from the time of diagnosis of HCM to the most recent follow-up. SCD was defined as witnessed SCD with or without documented ventricular fibrillation or death within 1 h of new symptoms or nocturnal deaths with no antecedent history of worsening symptoms (12). Successful resuscitation from ventricular fibrillation or ventricular tachycardia during follow-up and appropriate ICD shock therapy were considered equivalent to SCD (13, 14, 15, 16).

ECGs were reviewed blindly by 4 cardiologists (ML and LRL in adults, JPK and GN in children); disagreements were resolved by a third blinded reviewer (MMA); when original tracings were unavailable, data were obtained from the ECG description and interpretation in the medical record at the time of clinical evaluation. ECG classification was based on the presence or absence of abnormalities associated with HCM. Specifically: LV hypertrophy by Sokolow-Lyon criteria (SV1+RV5/6 >35 mV) in pediatric cases, and by Sokolow-Lyon or Cornell criteria (RaVL+SV3 >28 mV in men and >22 mV in women) in adults; and abnormal Q waves, and repolarization abnormalities.

Pediatric patients were defined as having been age <18 years at first evaluation. For the purposes of this analysis, patients were considered to have undergone CMR when they were scanned <2 years before the end of follow-up. Hypertension was defined as presence of the diagnosis on medical record and ongoing treatment with at least 1 antihypertensive agent.

### Statistical analysis

Statistical analyses were performed using IBM SPSS Statistics version 24.0 (IBM Corp., Armonk, New York) and STATA version 12. For descriptive statistics, variables are expressed as median (interquartile range [IQR]) or counts and percentages, as appropriate. The frequency of categorical variables was compared with chi-square or Fisher exact test as appropriate and continuous variables were compared using Student’s *t-*test (2-tailed, unpaired samples), Mann-Whitney *U* test, or Kruskal-Wallis analysis of variance. HCM penetrance was estimated using the Kaplan-Meier method with follow-up as time variable, abnormal ECG was analyzed as a time-varying covariate; groups were compared using the log-rank test. The 5 age categories used for Kaplan-Meier analysis were established based on clinically significant cutoffs. Hazard ratios were obtained by univariable and multivariable analyses using a Cox proportional hazards model. The proportional hazards assumption was verified using Schoenfeld residuals (17). The final Cox multivariable model was stratified by CMR because it violated the proportional hazards assumption as a covariate.

## Results

A total of 583 P/LP variant carriers from 307 families were evaluated; 267 (45.8%) were diagnosed with HCM at first evaluation and were excluded from the study. Supplemental Figure 1 shows the distribution by age of those diagnosed with HCM at first evaluation. A further 31 subjects underwent a single screening visit and were also excluded. The final study cohort therefore consisted of 285 individuals from 156 families who did not fulfill diagnostic criteria for HCM at first evaluation; 141 (49.5%) were male and median age at first evaluation was 14.2 years (IQR: 6.8 to 31.6 years); 167 subjects (58.6%) were aged <18 years (including some who have been previously reported [9]).

The frequency of causal genes was as follows: *MYBPC3* 123 individuals (43.2%), *MYH7* 69 individuals (24.2%), *TNNI3* 39 individuals (13.7%), *TNNT2* 34 individuals (11.9%), *TPM1* 9 individuals (3.2%), *MYL2* 6 individuals (2.1%), *ACTC1* a single individual (0.4%), and *MYL3* none. In addition, 4 individuals (1.4%) carried multiple P/LP variants (*MYBPC3* and *TNNI3* in 3, *MYH7* and *TNNT2* in 1). Supplemental Table 1 reports the variant details for the study cohort. By family, the frequency of causal genes was as follows: *MYBPC3* 71 families (45.5%), *MYH7* 44 families (28.2%), *TNNI3* 17 families (10.9%), *TNNT2* 15 families (9.6%), *TPM1* 3 families (1.9%), *MYL2* 3 families (1.9%), *ACTC1* one family (0.6%), *MYL3* none; 2 families (1.3%) carried multiple P/LP variants.

**Figure 1:**
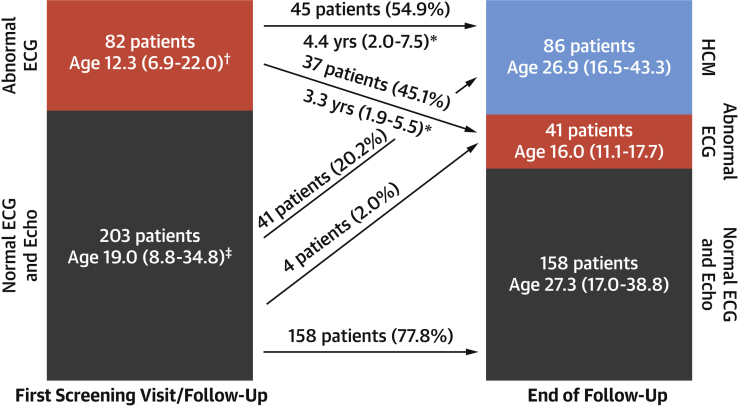
Phenotype in the Study Cohort During Follow-Up More than one-half of the subjects with an abnormal electrocardiogram (ECG) went on to develop overt hypertrophic cardiomyopathy (HCM). Those with an abnormal ECG but no overt HCM at the end of follow-up were younger, suggesting they will probably go on to develop overt HCM, although follow-up time from the first abnormal ECG was not clearly different between the 2 groups (∗p = 0.794). Ages reported are median (interquartile range). †At first abnormal ECG. ‡At first screening visit.

After a median follow-up of 8.0 years (IQR: 4.0 to 13.3 years), 86 patients (30.2%) reached the primary study endpoint; 41 (14.4%) had an abnormal ECG but did not fulfill diagnostic criteria and 158 (55.4%) had a normal ECG and echocardiogram (Figure 1). The 3 groups differed by genotype, sex, and age, with a higher male prevalence among those who developed overt HCM, and younger age in individuals with only an abnormal ECG compared with those with a normal phenotype and those with overt HCM (Table 1). Supplemental Figure 2 shows the phenotype at the end of follow-up according to age.

**Table 1:** Study Cohort Characteristics by Phenotype at the End of Follow-Up

	Normal ECG and Echo (n = 158)	Abnormal ECG Only (n = 41)	HCM (n = 86)	p Value
Genotype				
MYBPC3	76 (48.1)	9 (22.0)	38 (44.2)	<0.001
MYH7	28 (17.7)	16 (39.0)	25 (29.1)	
MYL2	4 (2.5)	0 (0.0)	2 (2.3)	
TNNI3	32 (20.3)	3 (7.3)	4 (4.7)	
TNNT2	11 (7.0)	12 (29.3)	11 (12.8)	
TPM1	5 (3.2)	1 (2.4)	3 (3.5)	
ACTC1	0 (0.0)	0 (0.0)	1 (1.2)	
Multiple mutations	2 (1.3)	0 (0.0)	2 (2.3)	
Age, yrs	16.9 (7.5–32.6)	6.9 (3.5–12.6)	16.0 (9.2–35.0)	<0.001
Male	72 (39.3)	24 (51.1)	55 (61.8)	0.002
Follow-up duration, yrs	9.1 (3.3–13.7)	6.9 (3.5–10.5)	7.7 (5.4–11.6)	0.276
Hypertension	5 (3.2)	1 (2.4)	7 (8.1)	0.206
CMR	75 (47.5)	20 (48.8)	50 (58.1)	0.270

Values are n (%) or median (interquartile range). p value is for overall comparison.

CMR = cardiac magnetic resonance; ECG = electrocardiogram; HCM = hypertrophic cardiomyopathy (i.e., fulfills diagnostic criteria).

Overall, HCM penetrance at 15 years follow-up was 46% (95% confidence interval [CI]: 38% to 54%) and was greater in male than in female individuals (58%; 95% CI: 47% to 70% vs. 33%; 95% CI: 24% to 46%) at 15 years follow-up (Central Illustration). Table 2 reports the characteristics of the study cohort by sex.

**Central Illustration:**
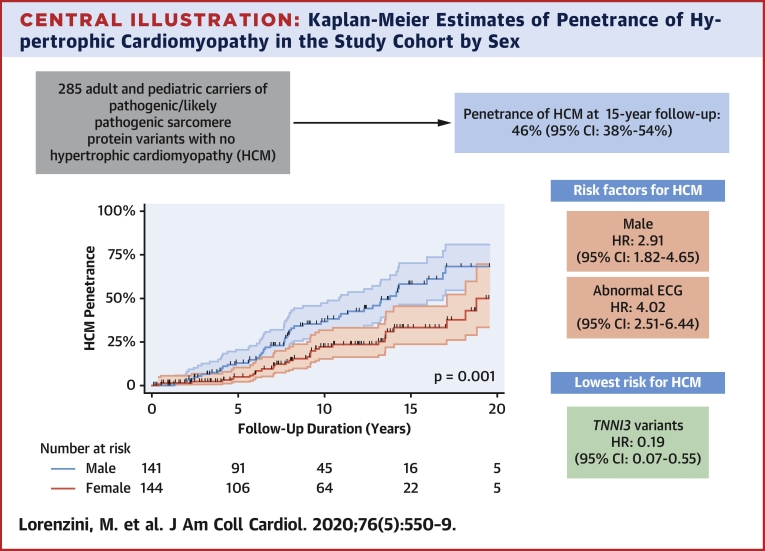
Kaplan-Meier Estimates of Penetrance of Hypertrophic Cardiomyopathy in the Study Cohort by Sex Male sex and abnormal electrocardiogram are risk factors for penetrance of hypertrophic cardiomyopathy (HCM) in carriers of pathogenic/likely pathogenic variants in sarcomere genes, while *TNNI3* variants are protective.

**Table 2:** Characteristics of the Study Cohort Stratified by Sex

	Male (n = 141)	Female (n = 144)	p Value
Genotype			
MYBPC3	62 (44.0)	61 (42.4)	0.581
MYH7	30 (21.3)	39 (27.1)	
MYL2	3 (2.1)	3 (2.1)	
TNNI3	17 (12.1)	22 (15.3)	
TNNT2	21 (14.9)	13 (9.0)	
TPM1	5 (3.5)	4 (2.8)	
ACTC1	0 (0.0)	1 (0.7)	
Multiple mutations	3 (2.1)	1 (0.7)	
Age, yrs	12.6 (6.8–28.5)	16.2 (6.7–35.6)	0.257
Follow-up duration, yrs	7.7 (3.3–12.3)	9.2 (4.6–13.6)	0.027
Abnormal ECG during follow-up	44 (31.2)	38 (26.4)	0.369
Hypertension	5 (3.5)	8 (5.6)	0.416
CMR	71 (50.4)	74 (51.4)	0.861
Diagnostic CMR, nondiagnostic echo	10 (7.1)	6 (4.2)	0.283

Values are n (%) or median (interquartile range). p value is for comparison.

Abbreviations as in Table 1.

**Figure 2:**
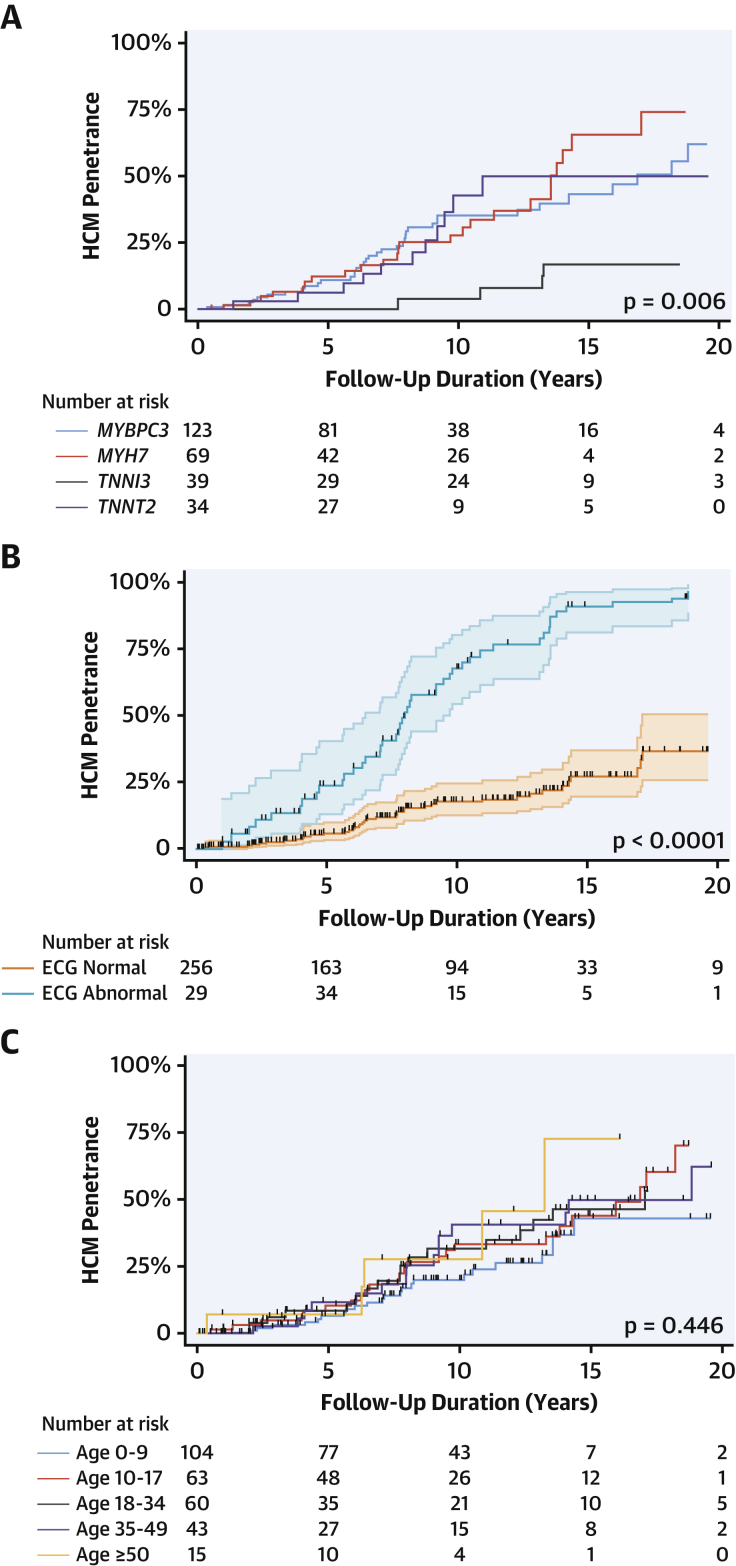
Kaplan-Meier Estimates of Penetrance of HCM in the Study Cohort By Causal Gene, ECG Phenotype, and Age at First Evaluation Penetrance is lowest in subjects with *TNNI3* variants, compared to *MYBPC3, MYH7,* and *TNNT2***(A)**, was similar in adult and pediatric subjects **(C)**, and an abnormal ECG was a strong predictor of subsequently developing overt HCM **(B)**. Abbreviations as in Figure 1.

At 15 years follow-up, estimated HCM penetrance by causal gene was as follows: *MYBPC3* 43% (95% CI: 32% to 57%), *MYH7* 66% (95% CI: 47% to 83%), *TNNI3* 17% (95% CI: 7% to 39%), *TNNT2* 50% (95% CI: 30% to 74%), *TPM1* 42% (95% CI: 11% to 92%), and multiple variants 63% (95% CI: 19% to 99%) (Figure 2A).

Supplemental Table 2 reports the characteristics of the study cohort by age at first evaluation; penetrance was similar in the pediatric and adult subgroups (Figure 2C).

At the time of diagnosis of HCM, 60 (72.3%) of 83 patients had an abnormal ECG (ECG data not available for 3 patients). Of these, 45 (54.2%) of 83 had an abnormal ECG before fulfilling the diagnostic criteria and this was first documented a median 4.4 years (range: 2.0 to 7.5 years) prior (range 0.2 to 13.1 years) (Figure 1). An abnormal ECG was a strong risk factor for a subsequent diagnosis of HCM (Figure 2B).

**Figure 3:**
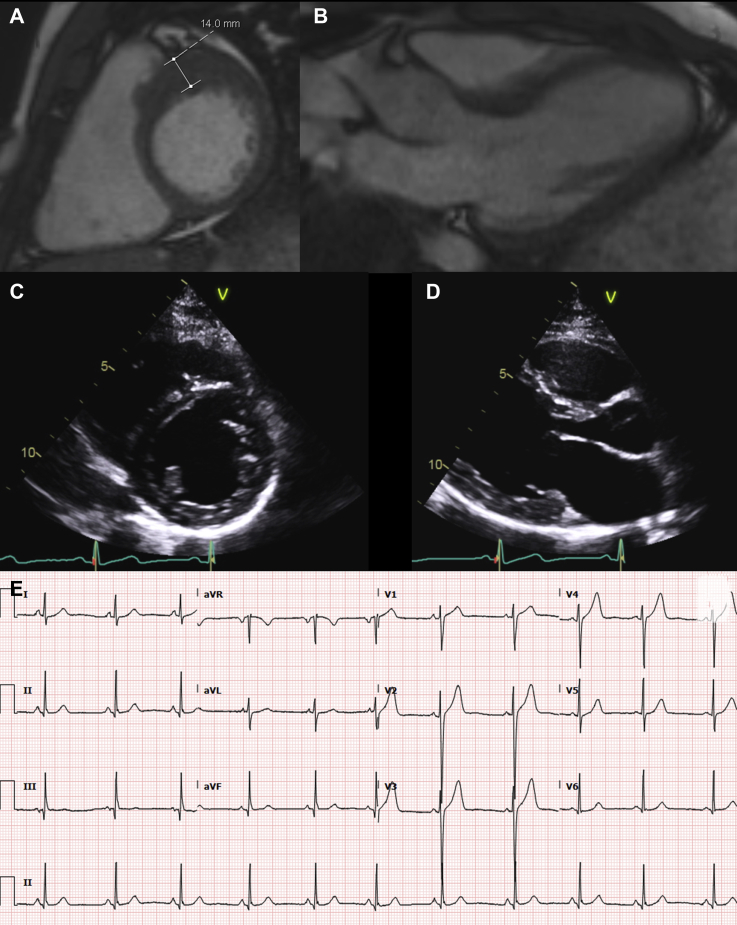
Utility of CMR for Screening Localized left ventricular hypertrophy involving the basal antero-septum and anterior wall in a 23-year-old man with a missense *MYH7* mutation (p.Cys695Arg) with an electrocardiogram showing no clear pathological features **(E)**. The hypertrophy can be appreciated on cardiac magnetic resonance (CMR) **(A, B)**, but not on echocardiogram **(C, D)**.

Overall, 145 subjects (50.9%) underwent a CMR with no clear difference between those who developed HCM and those who did not (Table 1). Among those diagnosed with HCM who underwent CMR, 16 (32.0%) of 50 fulfilled criteria on CMR but not echocardiography; 50% of these individuals (n = 8) had a normal ECG at the time of HCM diagnosis (Figure 3).

A multivariable model stratified by evaluation with CMR demonstrated the independent predictive value of male sex and an abnormal ECG before the end of follow-up. Compared with patients with *MYBPC3*, individuals with *TNNI3* variants had a lower penetrance of HCM. Older age at first evaluation was also associated with an increased risk (Table 3).

**Table 3:** Univariable and Multivariable Predictors of HCM Penetrance With Cox Regression

Predictor	Univariable Analysis			Multivariable Analysis		
	HR	95% CI	p Value	HR	95% CI	p Value
Age (10 yrs)	1.11	0.98–1.26	0.106	1.31	1.12–1.52	<0.001
Genotype						
MYBPC3	1.00	–	–	1.00	–	–
MYH7	1.22	0.73–2.02	0.447	1.13	0.67–1.92	0.644
MYL2	0.69	0.17–2.86	0.607	0.83	0.19–3.57	0.801
TNNI3	0.22	0.08–0.62	0.004	0.19	0.07–0.55	0.002
TNNT2	0.97	0.49–1.90	0.927	0.67	0.34–1.33	0.249
TPM1	0.78	0.24–2.53	0.677	0.41	0.12–1.39	0.152
ACTC1	8.91	1.19–66.91	0.034	13.51	1.67–109.14	0.015
Multiple mutations	0.84	0.20–3.49	0.806	1.22	0.29–5.21	0.786
Male	2.03	1.32–3.14	0.001	2.91	1.82–4.65	<0.001
Abnormal ECG∗	3.11	2.03–4.76	<0.001	4.02	2.51–6.44	<0.001
CMR	1.38	0.89–2.12	0.148	Analysis stratified for CMR		
Hypertension	1.90	0.87–4.14	0.107	-		

CI = confidence interval; HR = hazard ratio; other abbreviations as in Table 1.

∗ At baseline or during follow-up.

### Clinical outcomes

No cases of death or aborted SCD were recorded in individuals who did not fulfill HCM criteria. Following the diagnosis of HCM, over a median follow-up of 4.1 years (2.3 to 9.0 years) (total 528.4 person-years), 2 patients died suddenly (1 with an ICD in situ that was not downloaded at the time of postmortem), 1 experienced aborted SCD (ventricular fibrillation) and was successfully resuscitated, and 2 patients received appropriate ICD shocks. One patient died following superior vena cava rupture during ICD extraction. The overall event rate was 1.1% per year.

## Discussion

Based on the analysis of this large cohort of pediatric and adult SP gene P/LP variant carriers, disease penetrance in relatives without disease at first evaluation is higher than previously described (2, 3, 4, 5, 6, 7, 8). SCDs (or equivalent) were recorded exclusively in patients with an established diagnosis of HCM.

Several small studies have prospectively evaluated disease penetrance in SP P/LP variant carriers (2, 3, 4, 5, 6, 7, 8), reporting estimates up to 18%. However, most reports were limited by small cohort size, and some lacked generalizability due to inclusion of a high percentage of subjects with founder *MYBPC3* variants (7,8). The greater diversity in affected genes in our study might explain the higher disease penetrance observed. The younger age at baseline evaluation compared with the other published series (4,7) may also be relevant, as older age at inclusion in a prospective study probably biases toward individuals less likely to develop HCM.

A male prevalence of approximately 60% is a constant finding in large HCM cohorts and, based on our findings, this could be explained by a higher penetrance of SP gene P/LP variants in male individuals. After adjusting for baseline differences (including genotype), imaging technique, and age, we found the risk of developing HCM to be 3 times higher in male individuals. This remains unexplained, as does the higher mortality that has been observed in female individuals in large HCM cohorts in spite of this male predominance (18, 19, 20).

An abnormal ECG was strongly associated with the subsequent development of HCM (Figure 2B) and was independent of genotype and sex. This confirms the important role of the ECG as a screening tool as well as the need for long-term surveillance in individuals with an abnormal ECG. However, a normal ECG did not exclude a diagnosis of HCM because more than 1 in 4 patients had no major ECG abnormalities when diagnosed with HCM.

Although the relationship between HCM and hypertension remains to be fully clarified, because of the young age of the study cohort, the prevalence of hypertension in this study was low and was not associated with increased HCM penetrance. These findings are in line with a previous mouse model that suggested the existence of independent cardiac remodeling pathways in HCM and hypertension (21).

This is the first large study on disease penetrance in SP carriers to compare echocardiography with CMR. CMR was not clearly associated with a more frequent diagnosis of HCM during follow-up, but a subset of patients fulfilled diagnostic criteria on CMR but not on echocardiography. This may be explained by suboptimal echocardiographic imaging, particularly in regions of the LV that are more challenging to image, for example, the basal anterior and anterolateral wall (22). It is noteworthy that a small number of cases who fulfilled diagnostic criteria on CMR but not on echocardiography also had a normal ECG (Figure 3).

The growing number of healthy SP P/LP variant carriers is one of the greatest logistical problems facing clinical services dedicated to the evaluation of families with HCM, but the optimal timing and interval of screening is still debated (23, 24, 25). This study supports the need for lifelong surveillance that should commence at a younger age than currently recommended by the major societies in Europe and North America (1,24). Our data also suggest that, following an initial negative screening visit, the timing of follow-up should not be tailored to age, but rather to sex, ECG findings, and causal gene. Although larger prospective studies are needed to confirm our findings and establish the optimal timing of cardiac imaging, following the documentation of P/LP variant carrier status, CMR should be considered at baseline in adults, adolescents, and children old enough to undergo CMR without general anesthesia. Although our data suggest that regular CMR scans also should be considered during follow-up, in the absence of a standardized protocol in this study, the optimal interval for repeat CMR cannot be established. Until dedicated studies become available, the timing should, therefore, be based on the estimated risk in the individual subject as well as cost and local availability of CMR. Our data confirm the expected incidence of serious complications following the diagnosis of HCM, but also provide reassurance with the absence of major adverse events in P/LP variant carriers without a diagnosis of HCM, in line with previous reports (2, 3, 4, 5, 6, 7, 8).

### Study limitations

This was a retrospective analysis and patients were evaluated over a long time period in the absence of a standardized protocol. Only half of the subjects in this study underwent CMR and this may have led to an underestimation of disease penetrance.

Selection bias toward individuals with higher disease penetrance cannot be excluded because families with multiple affected members were preferentially offered genetic testing.

Sex is not considered in current HCM diagnostic criteria, and this may have led to an underestimation of penetrance in female individuals who tend to have a lower body surface area and a lower normal LV mass even when indexed (25). Finally, because data on comorbidities and body mass index were not systematically collected for this study, it is impossible to comment on their possible contribution to the observed sex differences.

## Conclusions

Following a first negative screening, approximately 50% of SP P/LP variant carriers develop HCM over 15 years of follow-up and become prone to disease complications during long-term follow-up. Male sex and the presence of an abnormal ECG are associated with a higher risk of disease development. Regular CMR should be considered in long-term screening.

Perspectives **COMPETENCY IN MEDICAL KNOWLEDGE:** Nearly 50% of patients with sarcomere protein gene mutations associated with HCM who do not meet diagnostic criteria for HCM develop HCM over 15 years follow-up, and development of hypertrophy is more frequent in male individuals.

**TRANSLATIONAL OUTLOOK:** Future studies should address the pathophysiological mechanisms underlying the differential hypertrophic response in male and female carriers of sarcomere protein mutations.
