# Supplementary material for: Penetrance of Hypertrophic Cardiomyopathy in Sarcomere Protein Mutation Carriers
Source: J Am Coll Cardiol. 2020 Aug 4;76(5):550–9. doi: 10.1016/j.jacc.2020.06.011 (PMC7397507; doi:10.1016/j.jacc.2020.06.011)
Supplement: Supplemental Figures 1 and 2 and Supplemental Tables 1 and 2 [file mmc1.docx]

**SUPPLEMENTAL MATERIAL**


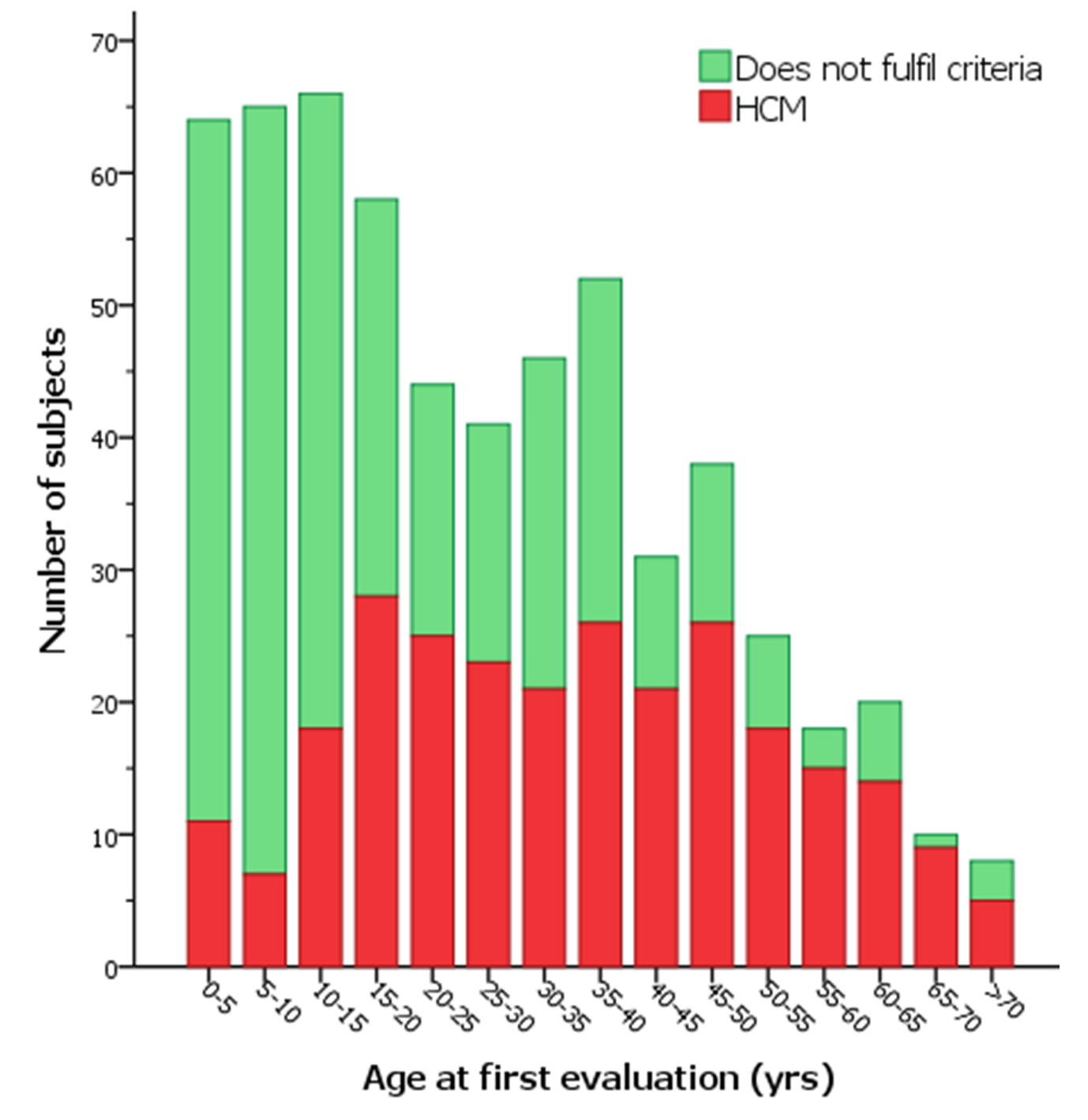


**Supplemental Figure 1.** Distribution by age of patients diagnosed with HCM at first evaluation and excluded from the study and those who did not fulfil criteria.


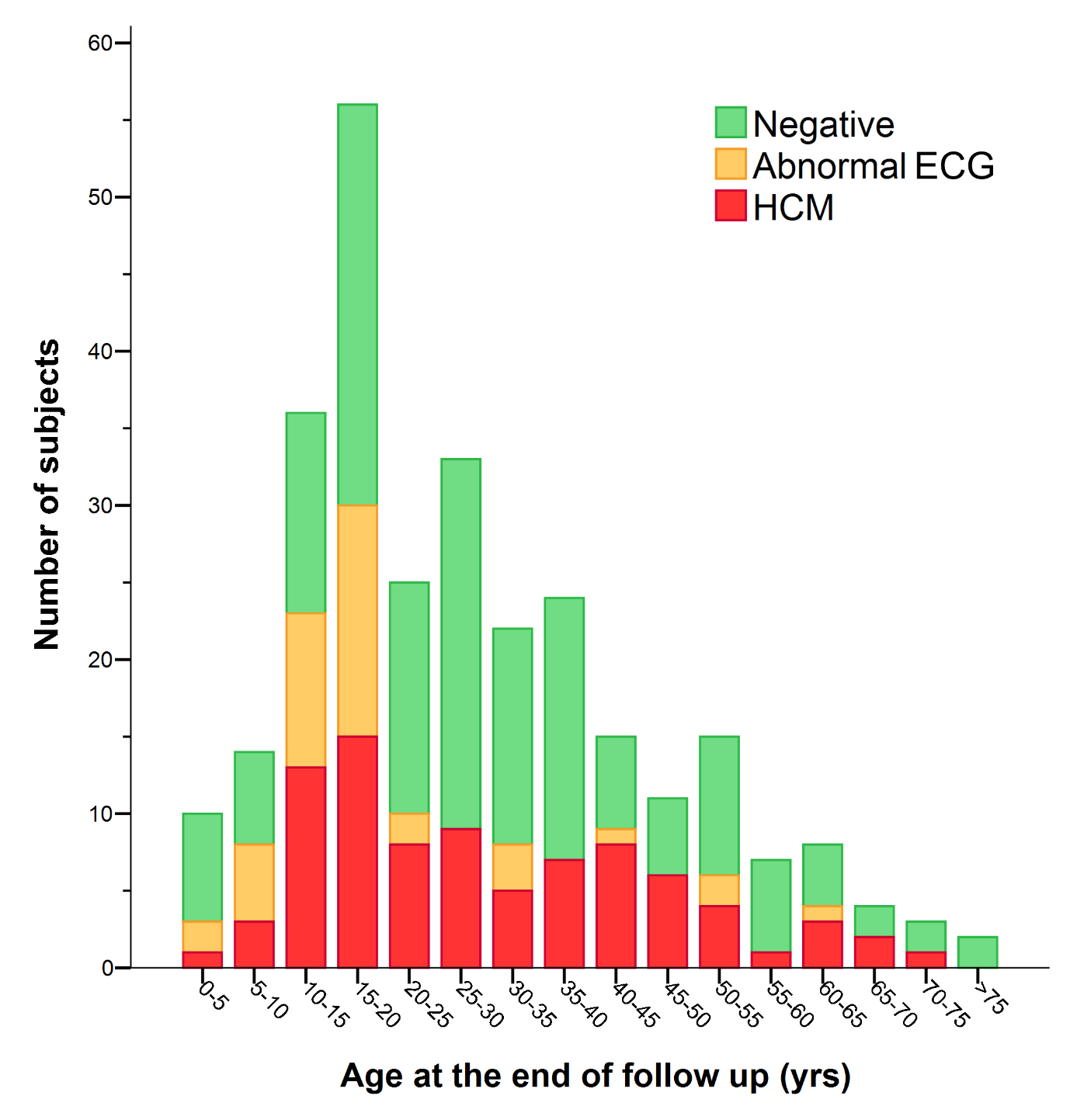


**Supplemental Figure 2.** Phenotype by age at the end of follow up in the study cohort.

**Supplemental Table 1: Variant details for the study cohort.**

| ***Gene*** | **Variant** | **Chromosomic nomenclature** | **Variant Type** | **ACMG variant classification** | **GnomAD frequency (MAF%)** | **Families** | **Subjects** |
| --- | --- | --- | --- | --- | --- | --- | --- |
| *MYBPC3* | p.Glu60AlafsTer49 | 11.9:g.47372898_47372908delTGCCCTCTGTG | Frameshift | Pathogenic | 0 | 2 | 3 |
| *MYBPC3* | p.Gly148Arg | 11.9:g.47371628C>T | Missense | Likely Pathogenic | 13/200,668 (0.006) | 1 | 1 |
| *MYBPC3* | p.Val219Leu | 11.9:g.47370092C>G | Missense | Pathogenic | 0 | 2 | 2 |
| *MYBPC3* | p.Glu258Lys | 11.9:g.47369975C>T | Missense | Pathogenic | 6/272,910 (0.002) | 1 | 1 |
| *MYBPC3* | c.821+2T>C | 11.9:g.47369406A>G | Splicing | Pathogenic | 0 | 1 | 1 |
| *MYBPC3* | c.927-9G>A | 11.9:g.47367930C>T | Splicing | Pathogenic | 2/217,270 (0.001) | 1 | 1 |
| *MYBPC3* | c.927-2A>G | 11.9:g.47367923T>C | Splicing | Pathogenic | 2/228,232 (0.001) | 1 | 6 |
| *MYBPC3* | p.Tyr333* | 11.9:g.47367849G>C | Nonsense | Pathogenic | 0 | 1 | 2 |
| *MYBPC3* | c.1090+1G>T | 11.9:g.47367757C>A | Splicing | Pathogenic | 1/248,814 (<0.001) | 1 | 1 |
| *MYBPC3* | p.His390MetfsTer16 | 11.9:g.47365099delG | Frameshift | Pathogenic | 0 | 1 | 4 |
| *MYBPC3* | c.1224-19G>A | 11.9:g.47364832C>T | Splicing | Pathogenic | 5/195,212 (0.005) | 1 | 1 |
| *MYBPC3* | c.1224-2A>G | 11.9:g.47364815T>C | Splicing | Pathogenic | 0 | 1 | 1 |
| *MYBPC3* | c.1227-13G>A | 11.9:g.47364709C>T | Splicing | Pathogenic | 4/270,442 (0.001) | 2 | 4 |
| *MYBPC3* | p.Arg495Gln | 11.9:g.47364269C>T | Missense | Pathogenic | 6/249,188 (0.002) | 3 | 7 |
| *MYBPC3* | p.Arg502Trp | 11.9:g.47364249G>A | Missense | Pathogenic | 13/280,632 (0.005) | 10 | 12 |
| *MYBPC3* | p.Arg502Gln | 11.9:g.47364248C>T | Missense | Pathogenic | 0 | 1 | 5 |
| *MYBPC3* | p.Gly531Arg | 11.9:g.47364162C>G | Missense | Likely Pathogenic | 5/278,462 (0.002) | 1 | 1 |
| *MYBPC3* | p.Glu542Gln | 11.9:g.47364129C>G | Missense | Pathogenic | 5/262,678 (0.002) | 5 | 6 |
| *MYBPC3* | c.1624+4A>T | 11.9:g.47364125T>A | Splicing | Pathogenic | 3/224,880 (0.001) | 4 | 6 |
| *MYBPC3* | p.Lys543ArgfsTer12 | 11.9:g.47363707delT | Frameshift | Pathogenic | 0 | 1 | 4 |
| *MYBPC3* | p.Arg597Gln | 11.9:g.47363542C>T | Missense | Likely Pathogenic | 5/166,884 (0.003) | 1 | 1 |
| *MYBPC3* | p.Asp610His | 11.9:g.47362758C>G | Missense | Likely Pathogenic | 8/253,926 (0.003) | 1 | 1 |
| *MYBPC3* | c.1928-2A>G | 11.9:g.47361343T>C | Splicing | Pathogenic | 0 | 1 | 1 |
| *MYBPC3* | p.Lys685ArgfsTer3 | 11.9:g.47361191_47361215del | Frameshift | Pathogenic | 0 | 1 | 1 |
| *MYBPC3* | p.Pro699GlnfsTer55 | 11.9:g.47360930delG | Frameshift | Pathogenic | 0 | 4 | 6 |
| *MYBPC3* | p.Trp792ValfsTer41 | 11.9:g.47359281_47359282insC | Frameshift | Pathogenic | 3/172,018 (0.002) | 6 | 19 |
| *MYBPC3* | p.Arg810His | 11.9:g.47359115C>T | Missense | Likely Pathogenic | 12/249,184 (0.005) | 1 | 1 |
| *MYBPC3* | p.Arg820Trp | 11.9:g.47359086G>A | Missense | Pathogenic | 1/249,210 (<0.001) | 1 | 1 |
| *MYBPC3* | p.Ser871AlafsTer8 | 11.9:g.47357560delG | Frameshift | Pathogenic | 11/168,356 (0.006) | 1 | 2 |
| *MYBPC3* | p.Ala938GlyfsTer113 | 11.9:g.47356690_47356691insC | Frameshift | Pathogenic | 0 | 1 | 3 |
| *MYBPC3* | p.Arg943ter | 11.9:g.47356671G>A | Nonsense | Pathogenic | 3/247,124 (0.001) | 1 | 2 |
| *MYBPC3* | p.Pro955ArgfsTer95 | 11.9:g.47356633_47356634delAG | Frameshift | Pathogenic | 1/264,126 (<0.001) | 2 | 3 |
| *MYBPC3* | p.Gln969Ter | 11.9:g.47356593G>A | Nonsense | Pathogenic | 0 | 1 | 2 |
| *MYBPC3* | p.Lys1055Ter | 11.9:g.47355135T>A | Nonsense | Pathogenic | 0 | 3 | 5 |
| *MYBPC3* | p.Trp1098Ter | 11.9:g.47354781C>T | Nonsense | Pathogenic | 0 | 1 | 1 |
| *MYBPC3* | p.Phe1159LeufsTer11 | 11.9:g.47354380_47354381insATAA | Frameshift | Pathogenic | 0 | 1 | 2 |
| *MYBPC3* | c.3491-2A>T | 11.9:g.47354255T>A | Splicing | Pathogenic | 0 | 1 | 2 |
| *MYBPC3* | p.Cys1202LeufsTer35 | 11.9:g.47354139delC | Frameshift | Pathogenic | 0 | 1 | 1 |
| *MYH7* | p.Ser271Pro | 14.8:g.23900194A>G | Missense | Likely Pathogenic | 0 | 1 | 1 |
| *MYH7* | p.Ala355Thr | 14.8:g.23899059C>T | Missense | Pathogenic | 0 | 1 | 2 |
| *MYH7* | p.Ala355Ser | 14.8:g.23899059C>A | Missense | Pathogenic | 0 | 1 | 1 |
| *MYH7* | p.Arg403Trp | 14.8:g.23898488G>A | Missense | Pathogenic | 0 | 1 | 1 |
| *MYH7* | p.Arg403Leu | 14.8:g.23898487C>A | Missense | Pathogenic | 0 | 1 | 3 |
| *MYH7* | p.Val406Met | 14.8:g.23898479C>T | Missense | Likely Pathogenic | 0 | 1 | 1 |
| *MYH7* | p.Val411Ile | 14.8:g.23898464C>T | Missense | Pathogenic | 6/282874 (<0.00002) | 1 | 1 |
| *MYH7* | p.Arg442Cys | 14.8:g.23898247G>A | Missense | Likely Pathogenic | 6/282,878 (0.002) | 1 | 1 |
| *MYH7* | p.Arg453Cys | 14.8:g.23898214G>A | Missense | Pathogenic | 0 | 1 | 2 |
| *MYH7* | p.Ile478Asn | 14.8:g.23897854A>T | Missense | Likely Pathogenic | 0 | 1 | 1 |
| *MYH7* | p.Met493Leu | 14.8:g.23897810T>A | Missense | Likely Pathogenic | 0 | 1 | 1 |
| *MYH7* | p.Met515Thr | 14.8:g.23897743A>G | Missense | Likely Pathogenic | 0 | 1 | 1 |
| *MYH7* | p.Val606Met | 14.8:g.23896866C>T | Missense | Pathogenic | 2/282,868 (<0.001) | 1 | 2 |
| *MYH7* | p.Arg663His | 14.8:g.23896042C>T | Missense | Pathogenic | 4/282,842 (0.001) | 3 | 4 |
| *MYH7* | p.Cys695Arg | 14.8:g.23895252A>G | Missense | Likely Pathogenic | 0 | 1 | 1 |
| *MYH7* | p.Asn696Ser | 14.8:g.23895248T>C | Missense | Likely Pathogenic | 0 | 1 | 1 |
| *MYH7* | p.Gly716Arg | 14.8:g.23895189C>T | Missense | Pathogenic | 0 | 1 | 1 |
| *MYH7* | p.Arg719Gln | 14.8:g.23895179C>T | Missense | Pathogenic | 0 | 1 | 2 |
| *MYH7* | p.Arg721Lys | 14.8:g.23895173C>T | Missense | Pathogenic | 0 | 1 | 4 |
| *MYH7* | p.Arg723Cys | 14.8:g.23895023G>A | Missense | Pathogenic | 3/251,214 (0.001) | 2 | 3 |
| *MYH7* | p.Ser738Arg | 14.8:g.23894978T>G | Missense | Likely Pathogenic | 0 | 1 | 1 |
| *MYH7* | p.Gly741Arg | 14.8:g.23894969C>G | Missense | Pathogenic | 0 | 2 | 9 |
| *MYH7* | p.Gly741Trp | 14.8:g.23894969C>A | Missense | Pathogenic | 0 | 1 | 1 |
| *MYH7* | p.Ala797Thr | 14.8:g.23894525C>T | Missense | Pathogenic | 6/251,468 (0.002) | 4 | 8 |
| *MYH7* | p.Asn817Lys | 14.8:g.23894206G>T | Missense | Likely Pathogenic | 0 | 1 | 1 |
| *MYH7* | p.Lys847Glu | 14.8:g.23894118T>C | Missense | Pathogenic | 0 | 3 | 3 |
| *MYH7* | p.Met849Thr | 14.8:g.23894111A>G | Missense | Likely Pathogenic | 0 | 1 | 1 |
| *MYH7* | p.Arg870His | 14.8:g.23894048C>T | Missense | Pathogenic | 2/282,808 (<0.001) | 1 | 1 |
| *MYH7* | p.Met877Ile | 14.8:g.23894026C>G | Missense | Pathogenic | 0 | 1 | 1 |
| *MYH7* | p.Leu889His | 14.8:g.23893991A>T | Missense | Likely Pathogenic | 0 | 1 | 2 |
| *MYH7* | p.Asp906Gly | 14.8:g.23893321T>C | Missense | Pathogenic | 1/251,460 (<0.001) | 1 | 3 |
| *MYH7* | p.Glu924Lys | 14.8:g.23893268C>T | Missense | Pathogenic | 0 | 1 | 1 |
| *MYH7* | p.Glu927Lys | 14.8:g.23893259C>T | Missense | Pathogenic | 0 | 2 | 2 |
| *MYH7* | p.Glu949Lys | 14.8:g.23893193C>T | Missense | Likely Pathogenic | 0 | 1 | 1 |
| *TNNI3* | p.Gln130Arg | 19.9:g.55665558T>C | Missense | Likely Pathogenic | 1/247986 (0.000004) | 1 | 3 |
| *TNNI3* | p.Arg141Gln | 19.9:g.55665525C>T | Missense | Pathogenic | 1/31370 (0.00003) | 2 | 4 |
| *TNNI3* | p.Arg145Trp | 19.9:g.55665514G>A | Missense | Pathogenic | 3/280226 (0.00001) | 2 | 9 |
| *TNNI3* | p.Ala157Val | 19.9:g.55665477G>A | Missense | Pathogenic | 0 | 1 | 2 |
| *TNNI3* | p.Arg162Gln | 19.9:g.55665463G>A | Missense | Pathogenic | 10/249030 (0.00004) | 2 | 7 |
| *TNNI3* | p.Arg162Trp | 19.9:g.55665463G>A | Missense | Pathogenic | 10/249000 (0.00004) | 2 | 3 |
| *TNNI3* | p.Ser166Phe | 19.9:g.55665450G>A | Missense | Likely Pathogenic | 2/249054 (0.000008) | 1 | 1 |
| *TNNI3* | p.Arg186Gln | 19.9:g.55663278C>T | Missense | Pathogenic | 0 | 2 | 3 |
| *TNNI3* | p.Asp196Asn | 19.9:g.55663249C>T | Missense | Pathogenic | 2/280974 (0.00001) | 3 | 6 |
| *TNNI3* | p.Gly203Arg | 19.9:g.55663228C>G | Missense | Likely Pathogenic | 0 | 1 | 1 |
| *TNNT2* | p.Glu83Lys | 01.10:g.201334755C>T | Missense | Likely Pathogenic | 1/31400 (0.00003) | 1 | 1 |
| *TNNT2* | p.Asp86Tyr | 01.10:g.201334746C>A | Missense | Likely Pathogenic | 0 | 1 | 1 |
| *TNNT2* | p.Arg92Trp | 01.10:g.201334426G>A | Missense | Pathogenic | 2/282802 (0.000007) | 4 | 6 |
| *TNNT2* | p.Arg92Gln | 01.10:g.201334425C>T | Missense | Pathogenic | 0 | 1 | 2 |
| *TNNT2* | p.Arg92Leu | 01.10:g.201334425C>A | Missense | Pathogenic | 0 | 1 | 2 |
| *TNNT2* | p.Glu163del | 01.10:g.201332514_201332516delCTC | Inframe Deletion | Pathogenic | 0 | 2 | 10 |
| *TNNT2* | p.Glu163Lys | 01.10:g.201332507C>T | Missense | Likely Pathogenic | 0 | 1 | 1 |
| *TNNT2* | c.821+1G>A | 01.10:g.201328750C>T | Splicing | Pathogenic | 0 | 1 | 2 |
| *TNNT2* | p.Arg278Cys | 01.10:g.201328373G>A | Missense | Likely Pathogenic | 98/275570 (0.0003) | 4 | 9 |
| *TPM1* | p.Glu192Lys | 15.9:g.63353922G>A | Missense | Pathogenic | 0 | 2 | 7 |
| *TPM1* | p.Asp175Asn | 15.9:g.63353098G>A | Missense | Pathogenic | 5/282804 (0.00001) | 1 | 2 |
| *MYL2* | p.Arg58Gln | 12.11:g.111352091C>T | Missense | Pathogenic | 2/251438 (<0.00001) | 1 | 1 |
| *MYL2* | p.Gly87Ala | 12.11:g.111352004C>G | Missense | Pathogenic | 0 | 2 | 5 |
| *ACTC1* | p.Phe92del | 15.9:g.35085625_35085627delAAG | Inframe deletion | Likely Pathogenic | 0 | 1 | 1 |
| *MYBPC3 TNNI3* | p.Glu542Gln  p.Arg162Gln | 11.9:g.47364129C>G  19.9:g.55665462C>T | Splicing  Missense | Pathogenic  Pathogenic | 5/262678 (0.00001)  0 | 1 | 3 |
| *TNNT2 MYH7* | p.Arg278Cys  p.Asp239Asn | 01.10:g.201328373G>A  14.8:g.23900811C>T | Missense  Missense | Pathogenic Pathogenic | 98/275570 (0,0003) 1/251496 (0.000003) | 1 | 1 |

**Supplemental Table 2: Characteristics of the study cohort stratified by age at first evaluation.**

|  | **Age <18 yrs**  **(n=167)** | **Age ≥18 yrs**  **(n=118)** | **P value** |
| --- | --- | --- | --- |
| **Genotype:** *MYBPC3*  *MYH7*  *MYL2*  *TNNI3*  *TNNT2*  *TPM1*  *ACTC1*  Multiple mutations | 69 (41.3%)  44 (26.3%)  6 (3.6%)  16 (9.6%)  23 (13.8%)  6 (3.6%)  0  3 (1.8%) | 54 (45.8%)  25 (21.2%)  0  23 (19.6%)  11 (9.3%)  3 (2.5%)  1 (0.8%)  1 (0.8%) | 0.047 |
| **Male sex** | 89 (53.3%) | 52 (44.1%) | 0.125 |
| **Follow up duration (years)** | 8.7 [4.9-13.3] | 7.2 [2.5-13.3] | 0.042 |
| **Hypertension** | 0 | 13 (11.0%) | <0.001 |
| **CMR** | 86 (51.5%) | 59 (50.0%) | 0.803 |
| **Diagnostic CMR, non-diagnostic echo** | 6 (3.6%) | 10 (8.5%) | 0.078 |
| **Maximal wall thickness at diagnosis:** <13mm  13-14 mm  15-16 mm  17-18 mm  >18 mm | 13/47 (27.7%)  18/47 (38.3%)  10/47 (21.3%)  2/47 (4.3%)  4/47 (8.5%) | -  25/39 (64.1%)  7/39 (17.9%)  3/39 (7.7%)  4/39 (10.3%) |  |

CMR: cardiac magnetic resonance. P value is for comparison.
